# Prevention of canine ocular thelaziosis (*Thelazia callipaeda)* with a combination of milbemycin oxime and afoxolaner (Nexgard Spectra^®^) in endemic areas in France and Spain

**DOI:** 10.1051/parasite/2019001

**Published:** 2019-01-15

**Authors:** Wilfried Lebon, Jacques Guillot, Maria-Jesús Álvarez, José Antonio Bazaga, Marie-Laure Cortes-Dubly, Pascal Dumont, Marianne Eberhardt, Héctor Gómez, Olivier Pennant, Noémie Siméon, Frederic Beugnet, Lénaïg Halos

**Affiliations:** 1 Boehringer Ingelheim Animal Health, CRSV 805 Allée des Cyprès 01150 Saint-Vulbas France; 2 Unité de Parasitologie, Mycologie, Dermatologie, Ecole Nationale Vétérinaire d’Alfort 94704 Maisons-Alfort France; 3 Hospital Canis de Monforte Carretera Circunvalación S/N 27400 Monforte (Lugo, Galicia) Spain; 4 Clínica Veterinaria Bazaga Ronda Sur, 50 10300 Navalmoral de la Mata Cáceres (Extremadura) Spain; 5 Clinique Vétérinaire de Gabarret Avenue de Marcadieu 40310 Gabarret France; 6 Hospital Veterinario Abros Parque empresarial Pereiro de Aguiar Polígono 2 A – Parcela 32A 32710 Pereiro de Aguiar (Orense, Galicia) Spain; 7 Clinique Vétérinaire Fénelon 5 boulevard Fénelon 24380 Vergt France; 8 Clinique Vétérinaire Sanilhac Avenue du 19 mars 1962 24660 Notre-Dame de Sanilhac France; 9 Boehringer Ingelheim Animal Health 29 avenue Tony Garnier 69007 Lyon France

**Keywords:** *Thelazia callipaeda*, eyeworm, prevention, dog, milbemycin oxime, Europe

## Abstract

In the past decade, canine thelaziosis due to *Thelazia callipaeda* has been diagnosed in an increasing number of European countries, with endemic areas being identified. A multi-center field trial was conducted in endemic areas in France and Spain to evaluate the efficacy of monthly administrations of the oral milbemycin oxime/afoxolaner combination (NexGard Spectra^®^) for the prevention of *T. callipaeda* infection in at-risk dogs. A total of 79 dogs negative for *T. callipaeda* and with a clinical history of eyeworm infection in the past two years completed the study. Dogs were randomly allocated either to a negative control group (42 dogs) or to the NexGard Spectra^®^ treated group (37 dogs). All dogs were followed up for a 6-month period and assessed monthly for the presence of nematodes on the eyes and for the signs of ocular thelaziosis (e.g., conjunctivitis, keratitis, and ocular discharge). When the presence of nematodes was confirmed, the conjunctival fornix was flushed with a saline solution for parasite recovery and counting, and the dogs were treated appropriately. Recovered parasites were stored in 70% alcohol for subsequent morphological identification. During the course of the study, 57.1% (24/42) of the control dogs were diagnosed positive for *Thelazia* infection, which illustrates a high incidence rate of parasite infection. Conversely, no eyeworm was recovered from any of the 37 dogs that received NexGard Spectra^®^. All parasites sampled were confirmed to be *T. callipaeda*. This clinical field study demonstrated that monthly administrations of NexGard Spectra^®^ provided 100% preventive efficacy against canine thelaziosis.

## Abstract (fr)

Depuis une dizaine d’années, la thélaziose canine due à *Thelazia callipaeda* est diagnostiquée dans un nombre grandissant de pays européens où des zones d'endémie sont identifiées. Une étude multicentrique de terrain a été conduite dans plusieurs de ces zones endémiques en France et en Espagne afin d’évaluer l’efficacité de l’administration orale mensuelle de milbémycine-oxime en combinaison avec l’afoxolaner (NexGard Spectra^®^) pour la prévention des infestations à *T. callipaeda* chez des chiens à risque. Un total de 79 chiens négatifs pour *T. callipaeda* et avec des antécédents de thélaziose clinique dans les deux années précédentes ont participé à l’étude. Ces chiens ont été alloués de manière aléatoire à un groupe contrôle négatif (42 chiens) ou un groupe traité avec NexGard Spectra^®^ (37 chiens). Tous les chiens ont été suivis pendant une période de 6 mois et évalués mensuellement pour la présence de nématodes dans l’œil et de signes cliniques oculaires de thélaziose (conjonctivite, kératite, écoulement oculaire). Lorsque la présence de nématodes a été confirmée, le sac conjonctival a été rincé avec une solution saline afin de collecter et compter les parasites et les chiens ont reçu un traitement approprié. Les parasites collectés ont été conservés dans l’alcool à 70 % pour une identification morphologique ultérieure. Pendant la durée de l’étude, 57.1 % (24/42) des chiens du groupe contrôle ont été diagnostiqués positif pour une infestation par *Thelazia* ce qui correspond à une incidence élevée d’infestation parasitaire. Au contraire, aucun parasite n’a été retrouvé chez les 37 chiens ayant reçu NexGard Spectra^®^. L’ensemble des parasites collectés ont été identifiés comme étant *T. callipaeda.* Cette étude clinique de terrain démontre que l’administration mensuelle de NexGard Spectra^®^ prévient à 100 % la thélaziose canine.

## Introduction

Nematodes of the genus *Thelazia* (Spirurida, Thelaziidae), also called eyeworms, inhabit the orbital cavity and associated tissues of several species of warm-blooded animals [1]. For decades, the distribution of *T. callipaeda* Railliet and Henry, 1910 was confined to the far-east part of the European continent and Asia [1, 28]. However, at the end of the 20th century, autochthonous cases were recorded in Italy [27]. An ecological model predicted the spread of the parasite across Europe due to the potential wide distribution of the intermediate host, the male fruitfly *Phortica variegata* [18, 20, 22], and through infected dogs travelling to/from endemic regions [18, 25]. As predicted, the list of endemic countries has expanded from Italy to include most countries of mainland Europe [3–8, 10–16, 18, 20, 26, 31]. In addition, human cases have also been reported in endemic areas, indicating the importance of the nematode to public health [21].

In France, the first descriptions of canine thelaziosis in dogs were reported in 2007 from Dordogne in South-western France in foci that are now considered as endemic for the parasite [9, 14]. A recent questionnaire-based investigation conducted among French veterinary clinics revealed a new focus located south of the first one, in the Landes department [14].

In Spain, the first autochthonous canine case was reported in 2010 from the region of La Vera (Cáceres Province, western Spain) [7]. Since then, this geographical area has been considered endemic for canine thelaziosis with prevalence in dogs reaching 40% [16]. Recently, new foci have been identified in the Madrid area, and new cases continue to be reported from various locations in the country [13].

The clinical signs of thelaziosis are related to the presence of irritant foreign-bodies, i.e., the nematodes, in the conjunctival sac. Early signs including rheum and light ocular discharge are frequently followed by conjunctivitis, petechiae, oedema, keratitis and epiphora [13]. Treatment is based on the mechanical removal of the worms by flushing the eyes. Macrocyclic lactone-based products (i.e., topical moxidectin (2.5 mg/kg) or oral milbemycin oxime (0.5 mg/kg)) have been found to be efficacious therapeutics after single administration or two administrations one week apart, respectively [2, 17].

The purpose of the present study was to assess the efficacy of monthly oral administration of milbemycin oxime in combination with afoxolaner (NexGard Spectra^®^, Boehringer-Ingelheim Animal Health) for the prevention of canine thelaziosis under field conditions in France and Spain during the transmission period.

## Materials and methods

### Study design and ethics

The study was conducted in accordance with Good Clinical Practices as described in the International Cooperation on Harmonisation of Technical Requirements for Registration of Veterinary Medicinal Products, VICH Guideline 9 and with the VICH Guideline 7 “Efficacy of Anthelmintics: General Requirements” [29, 30]. All animals enrolled in the study were privately-owned dogs, and an informed consent and agreement was obtained from each owner before enrolment of the dog.

This negative controlled blinded field efficacy study used a randomised block design. Dogs were enrolled for a 6-month period between April and July 2017 in order to cover the seasonal transmission of the parasite, occurring from spring to fall.

### Study sites

**Figure 1.**
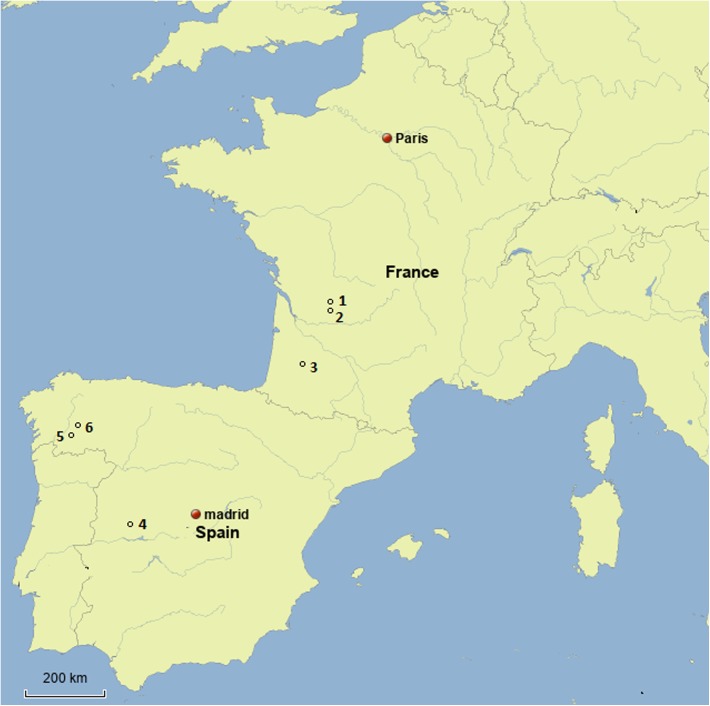
Map of the distribution of the six veterinary clinics involved in the study. 1: Notre-Dame de Sanilhac; France; 2: Vergt, France; 3: Gabarret, France; 4: Navalmoral de la Mata (Cáceres, Spain); 5: Pereiro de Aguiar (Orense, Galicia, Spain); 6: Monforte (Lugo, Galicia, Spain).

The study was conducted in six veterinary clinics in France and Spain (Fig. 1). In France, two veterinary clinics (in Notre-Dame de Sanilhac and Vergt) were located in Dordogne, the original focus for *T. callipaeda*, and the 3rd was located in the recently identified focus of the Landes department (Gabarret). In Spain, one clinic was located in the La Vera area in Navalmoral de la Mata, the original Spanish focus of canine thelaziosis, while two clinics were located in Galicia (North-western Spain, respectively in Monforte and Pereiro de Aguiar), an area not yet considered endemic for thelaziosis even though autochthonous cases have been reported by veterinarians for several years.

### Inclusion criteria

Client-owned dogs of both sexes, weighing at least 2 kg, ≥8 weeks of age and with a history of clinical diagnosis of thelaziosis during the previous two years were included. For the purpose of the study, the dogs had to be eyeworm-negative prior to the first treatment. Thus, all animals were dosed with a milbemycin oxime-based product labelled for the treatment of thelaziosis twice, one week apart prior to enrolment.

Physical examinations were performed at the pre-inclusion visit, on Day 0, and at each subsequent visit. During the veterinary consultations, all dogs underwent a physical examination, including an ophthalmological assessment for clinical signs of thelaziosis. At each visit, owners were also questioned about any abnormalities that may be related to the safety of the treatment. Dogs were managed under their normal conditions by their owners.

### Allocation and treatment

At inclusion, each dog was randomly allocated to one of the two treatment groups. Dogs allocated to group 1 (negative control) were treated orally six times at monthly intervals with a “placebo” product without anthelminthic activity (NexGard^®^, afoxolaner). Dogs in group 2 were treated orally six times at monthly intervals with NexGard Spectra^®^, (0.5 mg/kg milbemycin oxime and 2.5 mg/kg afoxolaner), according to the label instructions. Dogs were weighed prior to each treatment to determine the appropriate dosage. Neither personnel involved with assessment of efficacy nor owners were aware of which treatment was administered.

### Ocular examination and nematode count

At each visit, both eyes were examined for the presence of *Thelazia* nematodes. The conjunctival fornix (including underneath the third eyelid) was inspected for the presence of nematodes, and flushed with saline solution for parasite recovery if eyeworms were present. The collected nematodes were counted for each eye and stored in 70% ethanol for morphological identification.

In addition, clinical signs indicative of *Thelazia* infection (i.e., ocular discharge, conjunctivitis, keratitis, blepharospasm, and ulcer) were reported as present or absent. Any dog found positive for eyeworms was removed from the study and received appropriate curative treatment.

### Parasite identification and imaging

Identification of *T. callipaeda* was performed for all collected specimens using standard light microscopy at the Parasitology Unit of The National Veterinary School of Alfort (ENVA, France), according to morphological criteria [24].

In addition, two specimens were prepared for electron microscope imaging performed under a Scan Electron Microscope (SEM, FEI Quanta FEG 250) by the Centre Technologique des Microstructures, Lyon, France.

### Statistical analyses

The proportion of dogs free from *T. callipaeda* throughout the study was the efficacy criterion. A dog was considered positive as soon as an eyeworm was observed. The proportion of eyeworm-free dogs was compared between the treated and control groups using Fisher’s Exact Test for Count Data. The analysis was performed with SAS Version 9. The testing was two-sided at the significance level *α* = 0.05.

## Results

### Dog inclusion

A total of 88 dogs were enrolled in the study. It included 39 males and 49 females from various breeds, aged from 6 months to 14 years, and weighing 3.5–66.5 kg at the inclusion visit. The majority of these dogs (95.5%) were living in the countryside, 69.3% had free access to the outside, and 23.9% were housed outside.

Out of the 88 dogs, 79 completed the study and were included in the final statistical analysis, 42 dogs in control group 1 (negative control) and 37 dogs in treatment group 2 (Table 1). Nine dogs did not complete the study for various reasons, including four owner decisions, one accidental death, two protocol deviations, and two potential misdiagnoses (see below). No major adverse events related to treatments were observed during the study.

**Table 1.** Number of dogs completing the study in the 6 study sites and allocation to control or treated groups, parasite burden in positive animals, and incidence rate.

Site	Number of dogs per group		Number of eyeworm infected dogs per group	Range number of eyeworms per positive dog	Incidence rate
FR-01 Notre Dame de Sanilhac	Control group	10	6	1–4	60%
	Treated group	9	0	NA	0%
FR-02 Vergt	Control group	5	4	1–11	80%
	Treated group	6	0	NA	0%
FR-03 Gabarret	Control group	5	1	3	20%
	Treated group	4	0	NA	0%
ES-04 Caceres	Control group	7	5	1–3	71.4%
	Treated group	5	0	NA	0%
ES-05 Peihrero de Aguiera	Control group	5	1	15	20%
	Treated group	4	0	NA	0%
ES-06 Monteforte	Control group	10	7	2–16	70%
	Treated group	9	0	NA	0%
Total	Control group	42	24	1–16	57.1%
	Treated group	37	0	NA	0%

### Eyeworm detection

Results of the study are summarised in Table 1. Twenty-seven of the 88 enrolled dogs (30.7%) had eyeworms at the pre-inclusion visit (range: 1–55 adult nematodes) but tested eyeworm-negative following milbemycin oxime treatment before study initiation (Day 0).

**Figure 2.**
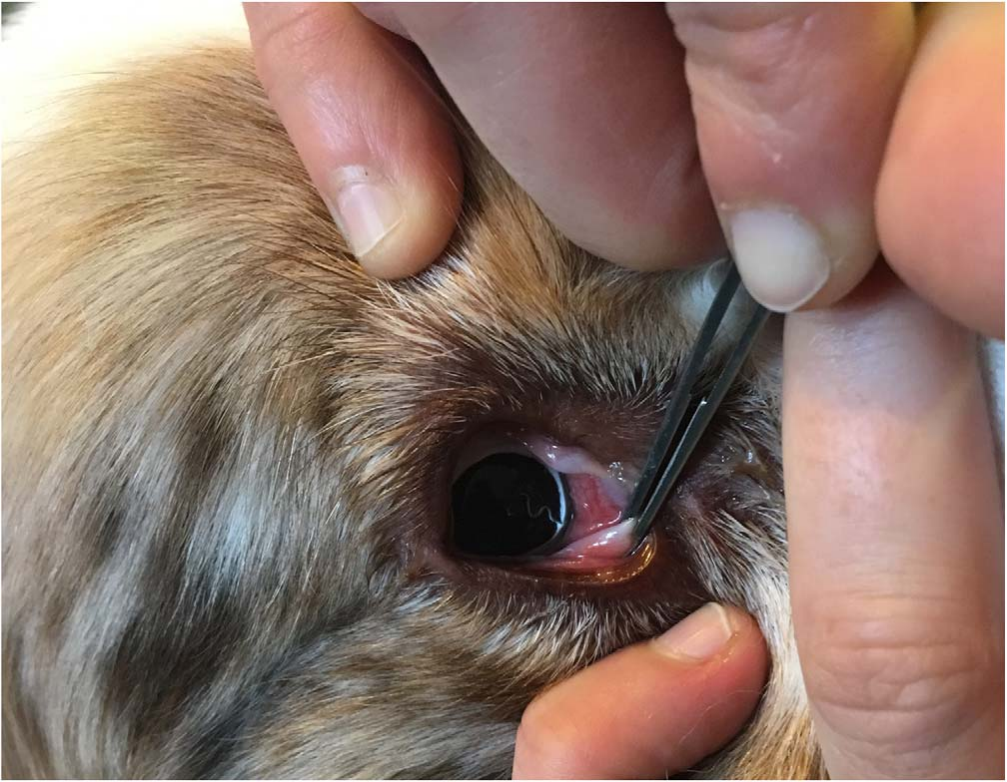
Presence of one single *Thelazia callipaeda* in the ocular cavity of one control dog; note conjunctivitis.

During the study, 26 dogs belonging to the negative control group tested eyeworm-positive (Fig. 2). This includes two dogs confirmed positive on Day 30. As the pre-patent period of *T. callipaeda* is considered to be 4–6 weeks [23], the decision was taken to withdraw these two dogs from the analysis because they were potentially harboring undetected worms at the inclusion visit. In total, 24/42 control dogs (57.1%) acquired eyeworm infection during the study.

In contrast, all 37 treated dogs remained negative throughout the study. Proportions of eyeworm-free dogs in the control group and the treated group was significantly different (*p* < 0.0001).

### Worm counts and clinical signs

Nematode counts in positive animals ranged from 1 to 16 during the course of the study, with most animals harboring ≤5 worms (76%) and demonstrating infection in one eye (63%). All *T. callipaeda* infections were associated with ocular signs, regardless of the number of worms involved. The most frequent signs observed in infected dogs included conjunctivitis (74% of the positive cases), epiphora (70%), pruritus (35%), purulent exudation (33%), and blepharospasm (20%).

### Parasite identification and imaging

**Figure 3.**
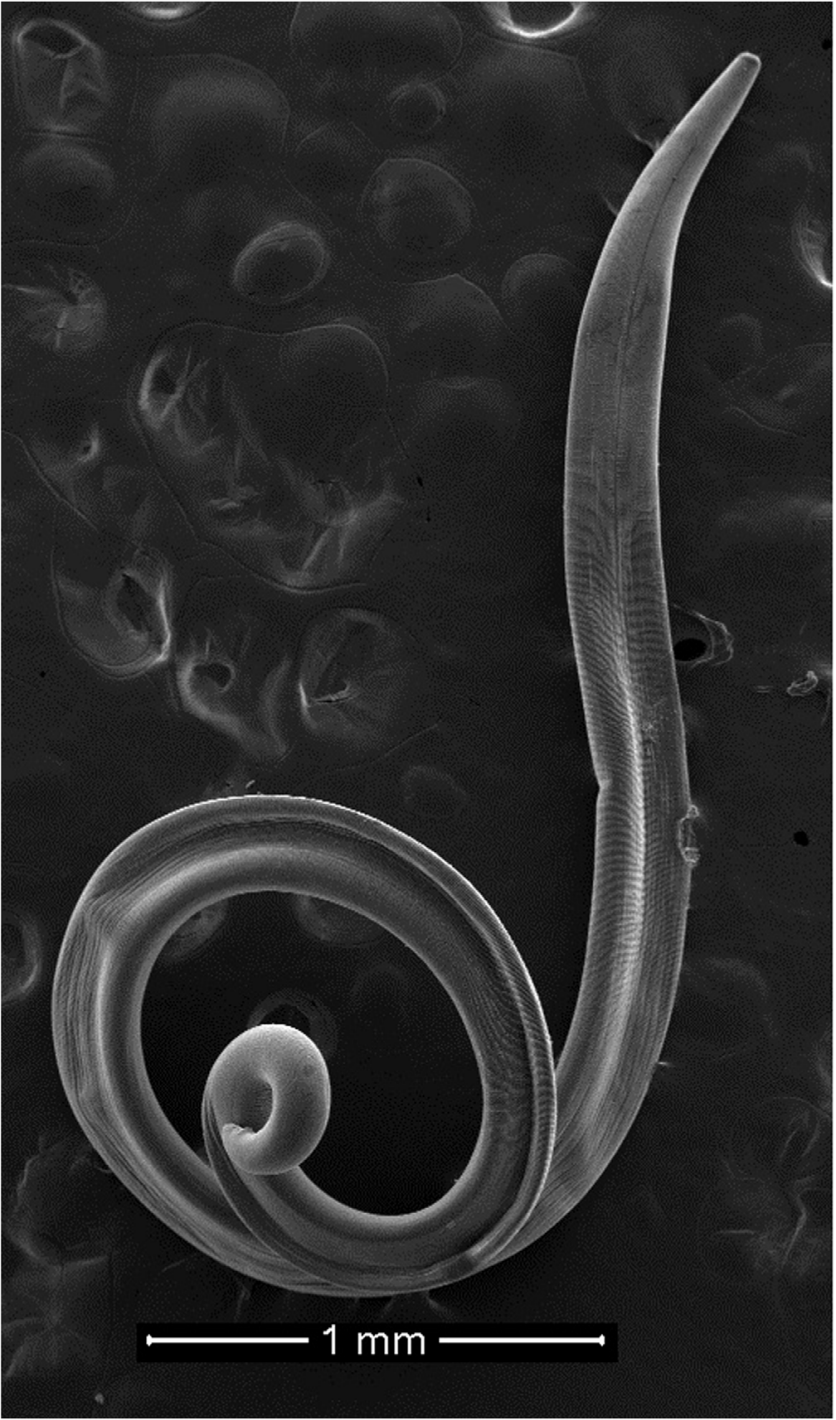
Male *Thelazia callipaeda*, scanning electron micrograph.

**Figure 4.**
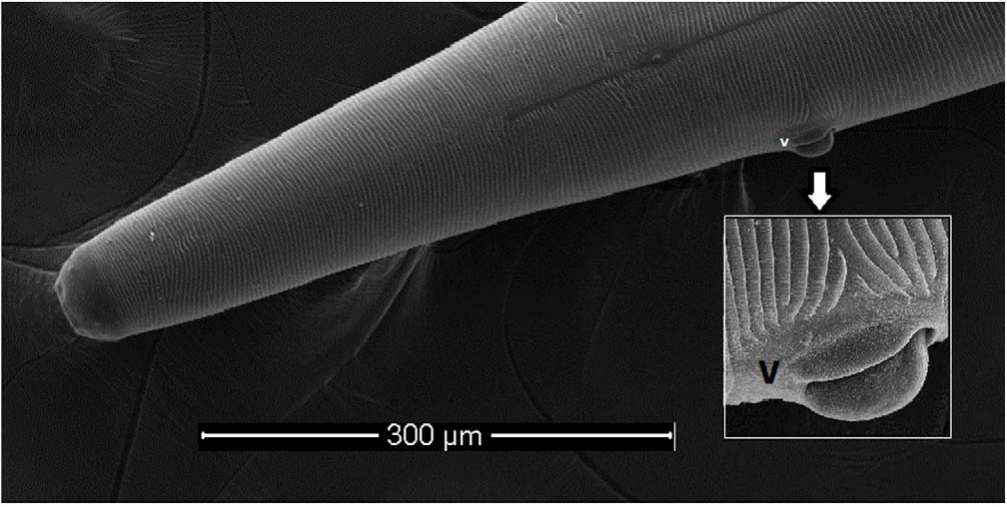
Female *Thelazia callipaeda*, anterior region, scanning electron micrograph. The transversally striated cuticle and the vulva (v) with vulvar flap are visible.

**Figure 5.**
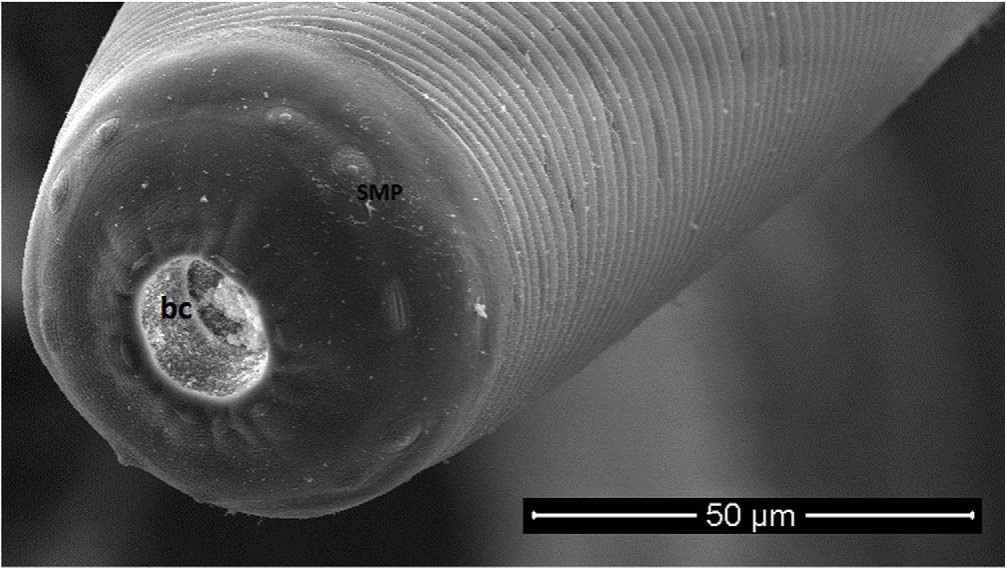
*Thelazia callipaeda*, scanning electron micrograph. Buccal capsule (bc) with an hexagonal shape of the mouth opening. Presence of sub-median papillae (smp).

All collected specimens were identified using light microscopy and confirmed to be *T. callipaeda* according to morphological criteria [23]. Briefly, they were small thin white nematodes with a transversally striated cuticle. Females were 10–15 mm in length and males were 7–10 mm in length. The position of the vulva located anterior to the esophagus-intestinal junction of the females and the presence of the post cloacal papillae in the males were characteristic of *T. callipaeda*. Electron microscope images of the two specimens observed under SEM are presented in Figures 3–5.

**Figure 6.**
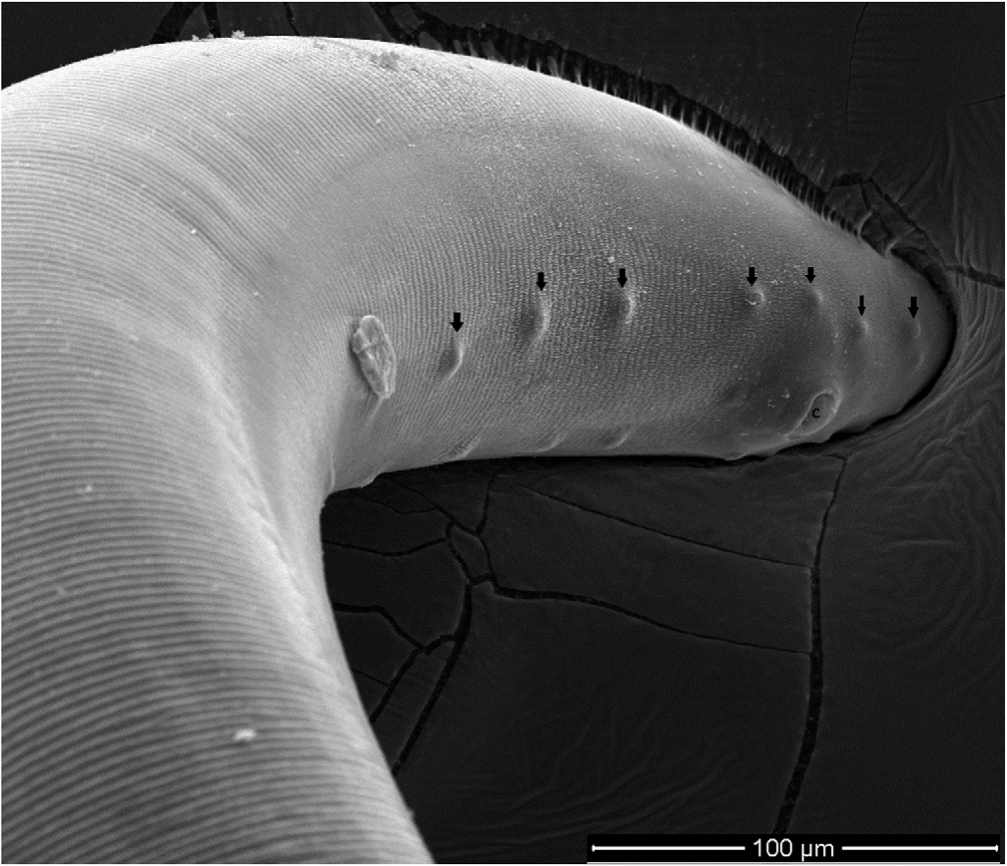
Male *Thelazia callipaeda*, posterior end, scanning electron micrograph. The pre-cloacal and post-cloacal papillae (arrows) are visible around the cloaca (c).

## Discussion

Cases of canine thelaziosis are increasingly reported throughout Europe [3–7, 9–15, 18, 19] and *T. callipaeda* is well established in some foci where prevalence in the dog population reaches 40%–60% [16, 22]. The present study was conducted either in locations where parasite occurrence was reported in the literature or in areas recently recognised as endemic. Because all dogs were free of worms at the beginning of the study, the number of infections observed in the control group allows us to estimate the six months incidence of the disease. The incidence rate observed was high and varied from 20% up to >80% depending on the location. Our results suggest an increase of incidence in the historical French foci in Dordogne (study sites 1 and 2). Indeed, a study conducted there five years ago in the same conditions reported an incidence of approximately 30% [9]. Moreover, these high infection rates raise concerns of a potential public health risk to the population in these areas.

Although most of the infected animals harboured only few eyeworms, all of them presented with clinical ocular thelaziosis. This is a far higher clinical incidence than that reported in previous studies: 15.4% (28/182) in Spain [16] or 45% in Portugal [11]. Interestingly, veterinarians from the study sites in France also reported ocular disorders (conjunctivitis, keratitis and epiphora) in dogs with a history of thelaziosis in the absence of nematodes, as well as an increase in clinical expression when eyeworms were present. This may be related to the development of hypersensitivity in dogs exposed to the parasite for several years.

In the early phase of infection, dogs often do not display clinical signs, and therefore the infection may go unnoticed by owners and veterinarians [12, 16, 17]. In addition, immature stages are unlikely to be noticed at the veterinary examination. In endemic areas, a preventive programme should be implemented in outdoor-living dogs, which are at risk of infection. With the parasite likely to extend its range to new geographies in Europe and its potential zoonotic risk, solutions combining safety, efficacy and ease-of-use are a significant improvement for veterinarians. In the present clinical field study, monthly administrations of a combination of milbemycin oxime and afoxolaner (NexGard Spectra^®^) provided complete preventive efficacy against canine thelaziosis.
